# The clinical and biological effects of childhood maltreatment in eating disorders: toward a distinct maltreated ecophenotype

**DOI:** 10.1192/j.eurpsy.2026.10397

**Published:** 2026-07-31

**Authors:** A. M. Monteleone

**Affiliations:** University of Campania L. Vanvitelli, Naples, Italy

## Abstract

**Introduction:**

Childhood maltreatment (CM) has been widely acknowledged as a risk factor for Eating Disorders (EDs). Although research has predominantly emphasized the role of sexual and physical abuse, literature has recently outlined the profound impact of childhood emotional abuse and neglect. However, the psychological and biological mechanisms underlying the associations between CM and EDs have not yet been clearly elucidated.

**Objectives:**

The objective of this presentation is summarizing findings that support the existence of a maltreated echo-phenotype of EDs.

**Methods:**

Data from our research group and from studies reported in the literature will be summerized and discussed.

**Results:**

Individuals with EDs and a history of CM show a more severe clinical presentation than those without CM. Findings regarding treatment response are mixed. Among the biological signatures characterizing individuals with EDs and CM, the main evidence suggests the presence of neuroanatomical alterations (namely, reduced integrity of gray and white matter and decreased cortical thickness) as well as abnormalities in the basal and acute functioning of the endogenous stress response system, as indicated by impaired cortisol and alpha-amylase patterns.

**Conclusions:**

The present findings suggest that, in addition to specific clinical features, CM is associated with distinct biological correlates in individuals with EDs, including neuroanatomical modifications, alterations in the endogenous stress response system, and changes in inflammation markers. This evidence supports the identification of a maltreated eco-phenotype of EDs, suggesting that CM acts as an etiological factor contributing to a distinct phenotypic expression of EDs that warrants specific attention to improve treatment outcomes.

**Disclosure of Interest:**

None Declared

